# Deubiquitinase USP37 enhances the anti-HIV-2/SIV ability of the host restriction factor SAMHD1

**DOI:** 10.1128/jvi.01858-24

**Published:** 2024-12-10

**Authors:** Wenzhe Cui, Hongfei Wang, Yuan Gao, Xue Zhang, Jingguo Xin, Zhaolong Li, Guangquan Li, Wenying Gao, Wenyan Zhang

**Affiliations:** 1 Center of Infectious Diseases and Pathogen Biology, Institute of Virology and AIDS Research, Key Laboratory of Organ Regeneration and Transplantation of The Ministry of Education, The First Hospital of Jilin University 154454 , Changchun, China; 2 Jilin Provincial Key Laboratory on Molecular and Chemical Genetics, The Second Hospital of Jilin University 117971 , Changchun, China; Icahn School of Medicine at Mount Sinai, New York, New York, USA

**Keywords:** SAMHD1, USP37, HIV/SIV, deubiquitination, host restriction factor

## Abstract

The Vpx protein encoded by HIV-2/simian immunodeficiency virus (SIV) can antagonize the restriction of the host intrinsic restriction factor, SAMHD1, in nondividing cells by promoting its polyubiquitination and subsequent degradation, thereby facilitating viral replication and immune evasion. However, the role of deubiquitinating enzymes (DUBs) in the dynamics of virus and host remains poorly understood. Here, we demonstrate that DUB USP37 significantly reverses the Vpx-mediated degradation of SAMHD1 in various HIV-2/SIV subtypes by interacting with SAMHD1 and removing its ubiquitin chains. Notably, USP37 deubiquitinates SAMHD1 by directly recognizing SAMHD1 rather than by targeting the E3 ubiquitin ligase. The deubiquitinase activity of USP37 and its ubiquitin interacting motifs are essential for the deubiquitination of SAMHD1, whereas the phosphorylation state of USP37 does not influence its activity. Additionally, USP37 enhances the suppression of the retrotransposition of LINE-1 elements by SAMHD1 via stabilizing SAMHD1. Our findings provide important evidence that enhancing the deubiquitinating activity of some DUBs results in the stability of the host restriction factor and might be a viable strategy against HIV/SIV infections.

**IMPORTANCE:**

SAMHD1 is a multifunctional protein, including restricting virus replication, maintaining genomic integrity through DNA repair, modulating the immune response by influencing the production of type I interferons and other cytokines, and affecting cancer cell proliferation and sensitivity to chemotherapy. However, HIV-2/simian immunodeficiency virus (SIV)-encoded Vpx and the host E3 ligase TRIM21 can induce the degradation of SAMHD1 via the ubiquitin–proteasome pathway. Therefore, it is necessary to find the strategy to stabilize SAMHD1. Our study demonstrates that the deubiquitinase USP37 reverses Vpx- and TRIM21-mediated degradation of SAMHD1, thereby inhibiting SIV replication and LINE-1 activity by stabilizing SAMHD1. Thus, we report a novel role of USP37, which represents a potentially useful target for the development of new drugs.

## INTRODUCTION

Many African nonhuman primate species are naturally infected with simian immunodeficiency viruses (SIVs) in the wild and in captivity (1, 2). Multiple cross-species transmissions of SIVcpz from chimpanzees and SIVsmm from sooty mangabeys (SMs) have led to the emergence of the current HIV-1 and HIV-2 epidemics, respectively (3, 4). Unlike HIV-1, which has a limited capacity to infect nondividing cells, such as dendritic cells and monocytes, HIV-2 and some SIV strains display a significantly enhanced ability to infect these cells. This increased infectivity is primarily attributed to the presence of the virion-associated Vpx accessory protein, which effectively counteracts the host’s intrinsic restriction mechanisms (5–7). The restriction factor inhibiting HIV/SIV infection in human primary myeloid cells was identified as the cellular sterile alpha motif (SAM) and HD domain-containing protein 1 (SAMHD1) (8). SAMHD1, with its extended HD domain, exhibits deoxynucleotide triphosphohydrolase activity and has been proposed to restrict HIV/SIV infection through multiple mechanisms (9). These include hydrolyzing intracellular dNTP pools (10–14) and modulating nucleic acid sensing pathways like the cGAS-STING pathway (15). However, the Vpx of HIV-2/SIVs assembles with CUL4A-DDB1 ubiquitin ligase through DCAF1 recruitment, specifically targeting SAMHD1 for proteasomal degradation. This process effectively mitigated the restriction of HIV-2/SIV infection (10). Our previous study also reported that SAMHD1 restricts enterovirus 71 (EV71), while EV71 overcomes the inhibitory effect of SAMHD1 by upregulating TRIM21 expression, which interacts and degrades SAMHD1 through the proteasomal pathway (16). Additionally, SAMHD1 also inhibits the transposition activity of long interspersed element 1 (LINE1) retrotransposon (17).

USP37, a member of the ubiquitin-specific protease (USPs) family, plays a significant role in cell cycle regulation and cancer progression via deubiquitination and stabilization of many essential proteins (18–21). The E3 ubiquitin ligase anaphase-promoting complex (APC/CDH1) substrate, cyclin A, is critical for G1/S transition. USP37 binds to CDH1 and removes degradative polyubiquitin chains, leading to the early accumulation of cyclin A in the G1 phase and accelerated entry into the S phase (18). In the context of DNA double-strand breaks (DSBs), USP37 and USP26 play pivotal roles in removing RNF168-induced ubiquitin conjugates from the BRCA1-A complex, without USP37 impairing DSB repair (22). Numerous previous studies have conclusively established a role for USP37 in stabilizing various oncogenes and promoting tumorigenesis (21, 23, 24). USP37 directly deubiquitinates and stabilizes c-Myc in lung cancer cells (23). USP37 is also involved in the deubiquitination of HIF2α in kidney cancer and regulates the stability of the oncogenic fusion protein PLZF/RARA through deubiquitination (21, 24). Ongoing research is increasingly exploring the role of cancer pathogenesis, with a growing focus on its potential as a therapeutic target in cancer treatment. However, the specific role USP37 plays in viral replication remains less explored, with limited studies currently present in the literature.

Ubiquitination and deubiquitination are dynamic equilibrium processes that occur in organisms. HIV-2/SIV Vpx recruits the host E3 ubiquitin ligase complex to degrade SAMHD1 via the proteasome pathway. A deubiquitinating enzyme (DUB) or several DUBs can reverse this process. In this study, we screened and identified USP37 as a DUB that inhibits Vpx-induced SAMHD1 ubiquitination and degradation, thereby suppressing wild-type SIV infectivity and retrotransposition activity of LINE1.

## RESULTS

### Screening of deubiquitinases inhibiting the downregulation of SAMHD1 induced by SIV

**Fig 1:**
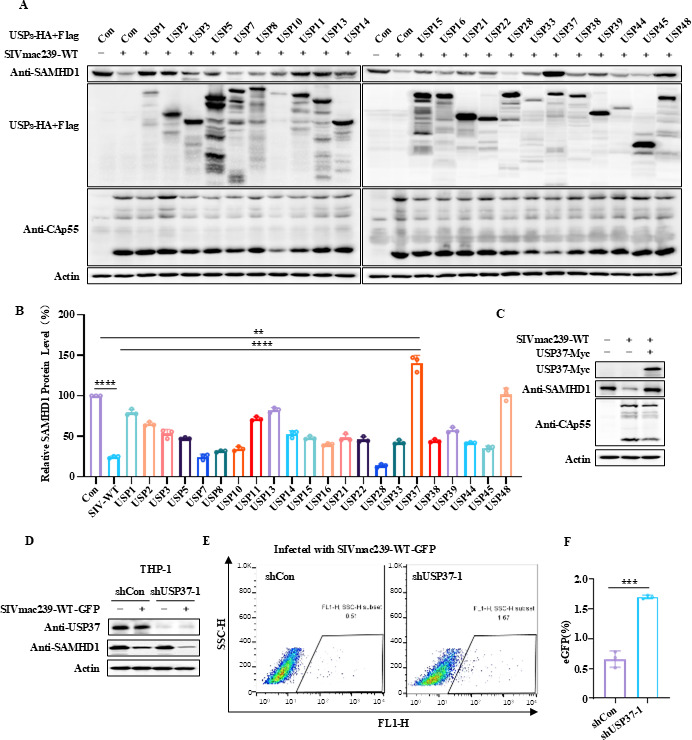
USP37 stabilizes SAMHD1 to inhibit SIV replication. (**A, B**) Effect of overexpression of deubiquitinase USPs on SIV-mediated SAMHD1 degradation. (**A**) The SIV expression vector SIVmac293-WT and the USP expression vector with the HA/Flag double tag were co-transfected into HEK293T cells. 48 h later, cells were harvested, and the protein expression was analyzed by immunoblotting (IB) using anti-SAMHD1 antibody targeting endogenous SAMHD1 protein, anti-HA antibody targeting USP protein, and CAp24 antibody targeting SIV viral proteins. Actin protein was used as a loading control. (**B**) SAMHD1 expression was quantified using ImageJ software in order to calculate the values relative to actin. (**C**) USP37 effectively stabilizes SIV-mediated SAMHD1 degradation. (**D**) Effect of USP37 silencing on SAMHD1 expression in SIVmac239-WT-infected THP-1 cell. USP37 knockdown THP-1 cells were differentiated into macrophages by 0.1 mM phorbol 12-myristate 13-acetate (PMA) treatment and infected with SIVmac239-WT viruses for 48 h. Endogenous USP37 and SAMHD1 were analyzed by immunoblotting. (**E, F**) USP37 knockdown THP-1 cells were differentiated into macrophages by 0.1 mM phorbol 12-myristate 13-acetate (PMA) treatment and infected with SIVmac239-WT-eGFP viruses for 48 h. (**E**) SIVmac239-eGFP-positive cells were isolated by flow cytometry. (**F**) Quantification of the eGFP-positive cells in (**D**). Data are means ± SD from *n* = 3 independent experiments. Statistical significance was analyzed using two-sided unpaired *t*-tests (***P < 0.001).

Previous studies have shown that HIV-2/SIV infection significantly downregulates the expression of host restriction factor, SAMHD1, to antagonize its antiviral function through the ubiquitination proteasomal pathway (5, 10, 11). To determine which DUB reverses the downregulation of SAMHD1 by HIV-2/SIV, we examined deubiquitinases that can reverse the degradation of SAMHD1 by SIVmac239. HEK293T cells were co-transfected with the SAMHD1 expression vector, SIVmac239 wild-type (SIVmac239-WT) expression vector plus the negative-control vector VR1012, or the indicated USP expression vectors and harvested 48 h later for IB analysis of endogenous SAMHD1 expression. We found that USP37 greatly increased the expression of SAMHD1 (Fig. 1A through C). Therefore, USP37 was selected for further analysis. To investigate the effect of USP37 on the stability of SAMHD1, USP37 was knocked-down in THP-1 cells expressing SAMHD1. The results showed that SIVmac239-WT-GFP virus infection decreased the expression of SAMHD1 in the USP37 knockdown group when compared to that in the control group (Fig. 1D). Accordingly, the infectivity of SIVmac239-WT-GFP was found to be significantly increased in the USP37 knockdown group (Fig. 1E and F), indicating that USP37-knockdown led to more unstable SAMHD1 upon SIVmac239 infection. Taken together, USP37 stabilizes SAMHD1 to inhibit SIVmac239-WT-GFP replication.

### USP37 reverses Vpx-mediated degradation of SAMHD1

**Fig 2:**
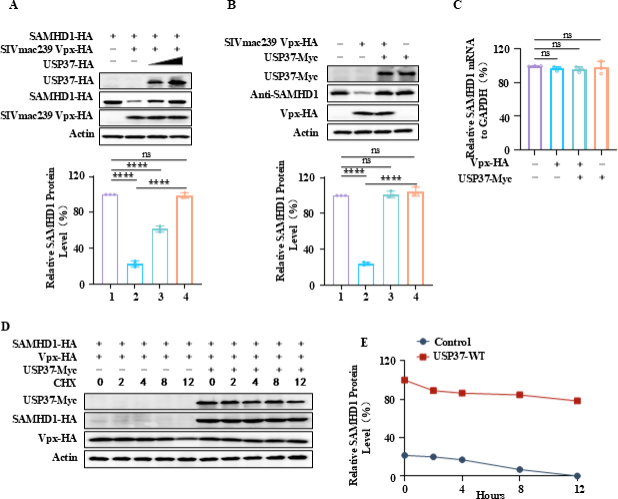
USP37 inhibits Vpx-mediated degradation of SAMHD1. (**A**) Overexpression of SIVmac-Vpx degrades exogenous SAMHD1-HA and is stabilized in a dose-dependent manner by USP37. Expression vector SAMHD1-HA, SIVmac-Vpx-HA, and USP37-HA were co-transfected into HEK293T cells. 48 h later, cells were harvested, and the protein expression was analyzed by IB (above). Quantiﬁcation of SAMHD1 expression was analyzed by ImageJ2X. SAMHD1 expression alone was normalized to 100% (below). (**B, C**) USP37 maintained endogenous SAMHD1 stability. (**B**) Expression vector SIVmac-Vpx-HA and USP37-HA were co-transfected into HEK293T cells. 48 h later, cells were harvested, and the protein expression was analyzed by IB (above). Quantiﬁcation of SAMHD1 expression was analyzed by ImageJ2X. SAMHD1 expression alone was normalized to 100% (below). (**C**) RT-PCR analysis of the effects of USP37 on SAMHD1 mRNA expression. (**D, E**) 24 h after transfection with the relevant plasmid, HEK293T cells were treated with 50 µg/mL CHX and harvested at the specified times. (**D**) The cell lysates were analyzed using IB. (**E**) SAMHD1 protein expression was performed using ImageJ2X.

Given that HIV-2/SIV Vpx hijacks the CRL4 (DCAF1) E3 ubiquitin ligase complex to degrade SAMHD1, thereby promoting viral infection, we investigated whether USP37 reverses SIVmac239 Vpx-mediated SAMHD1 degradation. Co-transfection of SAMHD1, SIVmac239, Vpx, and USP37 in HEK293T cells showed that USP37 inhibited the degradation of SAMHD1 by Vpx in a dose-dependent manner (Fig. 2A). USP37 also stabilized the expression of endogenous SAMHD1 in the presence of Vpx (Fig. 2B) without affecting the mRNA level of SAMHD1 (Fig. 2C). We then utilized cycloheximide (CHX), an inhibitor of eukaryotic translational elongation and protein synthesis, to treat SAMHD1-Vpx-transfected cells. CHX treatment led to a rapid time-dependent decline of SAMHD1 in the SAMHD1-Vpx group, whereas USP37 presence maintained stable SAMHD1 protein levels (Fig. 2D and E). Overall, these results indicated that USP37 reverses SIVmac239 Vpx-mediated SAMHD1 degradation.

### USP37 reverses the degradation of SAMHD1 mediated by different subtypes of HIV-2/SIV

**Fig 3:**
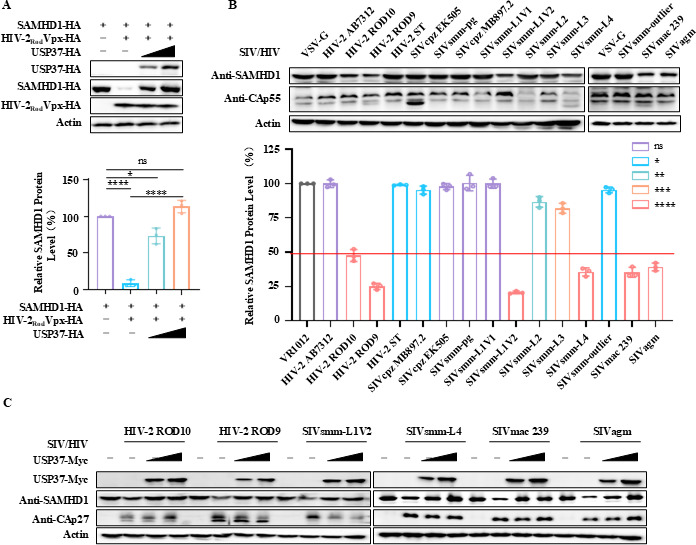
USP37 inhibits different subtypes of HIV-2/SIV-mediated degradation of SAMHD1. (**A**) USP37 overexpression inhibits degradation of SAMHD1 by HIV-2_Rod_ Vpx. Expression vector SAMHD1-HA, HIV-2_Rod_-Vpx-HA, and USP37-HA were co-transfected into HEK293T cells. 48 h later, cells were harvested, and the protein expression was analyzed by IB (above). Quantiﬁcation of SAMHD1 expression was analyzed by ImageJ2X. SAMHD1 expression alone was normalized to 100 (below). (**B**) Effect of 15 subtypes of SIV/HIV-2 on SAMHD1 expression. Different subtypes of the SIV/HIV-2 expression vector were co-transfected into HEK293T cells. 48 h later, cells were harvested, and protein expression was analyzed by immunoblotting using anti-SAMHD1 antibody targeting endogenous SAMHD1 protein and CAp24 antibody targeting SIV/HIV-2 viral proteins (above). SAMHD1 expression was quantified using ImageJ software to calculate the values relative to actin (below). (**C**) USP37 inhibited the degradation of SAMHD1 by HIV-2Rod9/10, SIVsmm-L1V2, SIVsmm-L4, and SIVagm viruses. Statistical significance was analyzed using two-sided unpaired *t*-tests (NS, not significant; *P < 0.05, **P < 0.01; ***P < 0.001, and ****P < 0.0001).

It has been reported that multiple subtypes of HIV-2 and SIV counteract SAMHD1 restriction via Vpx (10). We further determined that USP37 inhibits the degradation of SAMHD1 by HIV-2_Rod_ Vpx (Fig. 3A). To verify the effect of USP37 on the degradation of SAMHD1 by different subtypes of HIV-2 and SIV, we first examined whether these viruses induce the degradation of SAMHD1, with SIVmac239 serving as a positive control. The results showed that SIVmac239, HIV-2_Rod_9/10, SIVsmm-L1V2, SIVsmm-L4, and SIVagm degraded SAMHD1 (Fig. 3B). As expected, the presence of USP37 reversed the degradation of SAMHD1 by HIV-2Rod, SIVsmm-L1V2, SIVsmm-L4, and SIVagm viruses, as shown by the positive control SIVmac239 (Fig. 3C). Therefore, USP37 reverses the degradation of SAMHD1 by different HIV-2/SIV subtypes.

### USP37 interacts with SAMHD1 and removes polyubiquitination of SAMHD1

**Fig 4:**
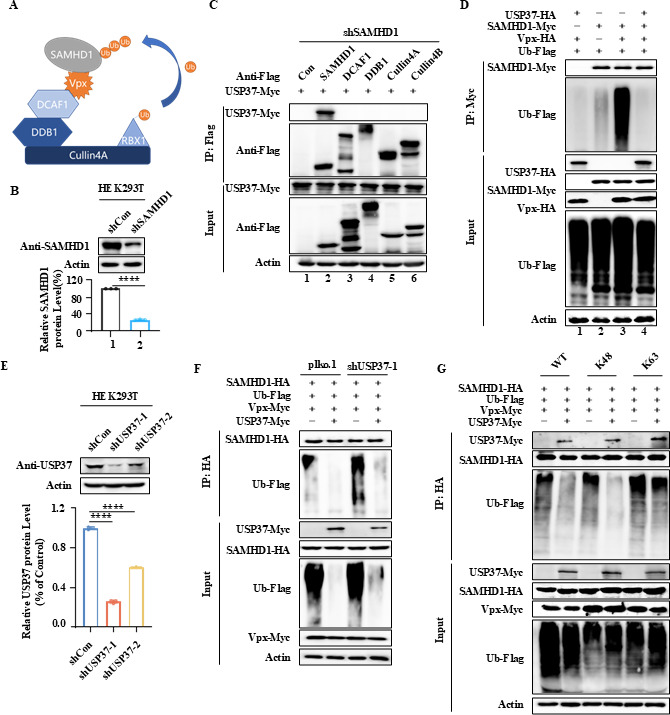
USP37 interacts with SAMHD1 and removes polyubiquitination of SAMHD1. (**A**) Diagram of the mechanism of Vpx degradation of the SAMHD1 model. (**B**) Immunoblotting (IB) detected SAMHD1 expression in 293T cells with a SAMHD1 knockout. The expression of the SAMHD1 protein was analyzed using IB (above), while the SAMHD1 mRNA expression level was measured using RT-PCR (below). (**C**) USP37 selectively interacts with SAMHD1 but not with the CRL4 E3 ubiquitin ligase complex. HEK293T cells were transfected with FLAG-tagged E3 ubiquitin ligase complex proteins and Myc-tagged USP37, and then treated with 10 mM MG132 for 12 h before harvesting. The cell lysates were then subjected to co-immunoprecipitation (co-IP) using anti-FLAG antibodies bound to agarose beads. Both lysates and co-IP samples were analyzed by immunoblotting using the respective antibodies. (**D**) USP37 inhibits the polyubiquitination of SAMHD1. HEK293T cells were transfected as instructed and treated with 10 mM MG132 for 12 h before collection. Subsequently, co-immunoprecipitation (co-IP) using anti-Myc antibodies and IB were performed. (**E**) USP37 expression was knocked down in HEK293T cells. The expression levels of the USP37 protein were analyzed using IB (above), and mRNA expression levels of USP37 mRNA were analyzed using RT-PCR (below). (**F**) USP37 silencing reduces SAMHD1 deubiquitination. SAMHD1 ubiquitination in USP37 silencing and control cells was analyzed by co-IP (with anti-FLAG) and IB. (**G**) USP37 deubiquitinates both K48- and K63-linked ubiquitin chains of SAMHD1. Data are presented as means ± SD from *n* = 3 independent experiments. Statistical significance was analyzed using two-sided unpaired *t*-tests (****P < 0.0001).

Vpx is an adaptor protein of the DCAF1-CUL4A/B E3 ubiquitin ligase that degrades SAMHD1 (Fig. 4A) (10, 25). To explore the mechanism of USP37-mediated SIVmac239 Vpx inhibition, we employed co-immunoprecipitation (co-IP) assay to determine whether USP37 interacts with SAMHD1 or the Vpx-CUL4A-DCAF1 E3 ubiquitin ligase complex in SAMHD1-knockdown HEK293T cells. The efficiency of SAMHD1-knockdown was confirmed by immunoblotting (Fig. 4B). Overexpression of SAMHD1-Flag, but not DDB1, DCAF1, CUL4A, or CUL4B, pulled down USP37 in precipitates (Fig. 4C), indicating a specific interaction between USP37 and SAMHD1. However, USP37 significantly reduced polyubiquitination of SAMHD1 induced by SIVmac239 Vpx (Fig. 4D, lanes 3 and 4). In contrast, USP37-knockdown did not deubiquitinate the SAMHD1 polyubiquitin chain, whereas USP37 overexpression removed the SAMHD1 polyubiquitin chain (Fig. 4F). The efficiency of USP37-knockdown was confirmed by immunoblotting (Fig. 4E). The K48- and K63-linked chains were the two most abundant linkage types. The K48-linked chain targets proteins for proteasomal degradation, whereas the K63-linked polyubiquitin chain is usually associated with the regulation of protein function and immune responses (26). SAMHD1 was ubiquitinated at both the K48 and K63 residues, whereas USP37 reduced both the K48-and K63-linked ubiquitination of SAMHD1 (Fig. 4G). Taken together, these results indicate that USP37 specifically interacts with SAMHD1 and removes polyubiquitination of SAMHD1.

### USP37 also deubiquitinates the polyubiquitin of SAMHD1 mutants that cannot be degraded by Vpx

**Fig 5:**
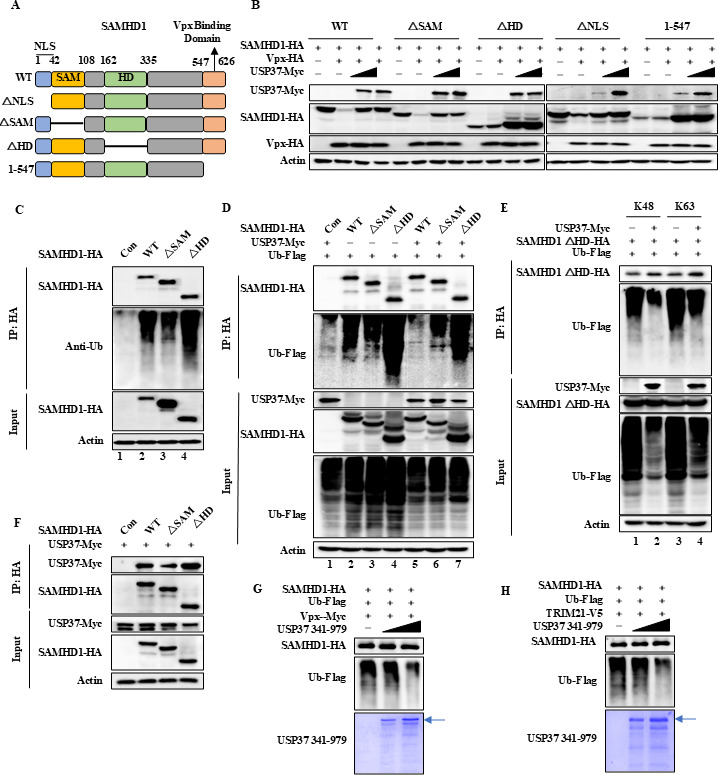
USP37 can deubiquitinate SAMHD1 mutants, even when they cannot be degraded by vpx. (**A**) Schematic representation of the SAMHD1 wild type and mutants used in the study. (**B**) HEK 293T was transfected, as indicated. After 48 h, cells were harvested, and protein expression was analyzed by IB. (**C**) HD structural domain deletion enhances the level of ubiquitination modification of SAMHD1. HEK293T cells transfected with the indicated expression vectors were treated with 10 mM MG132 for 12 h prior to harvesting. Cell lysates were co-IP by protein G agarose beads conjugated with anti-HA antibodies. Cell lysates and precipitated samples were analyzed using IB with the corresponding antibodies. (**D**) USP37 can deubiquitinate SAMHD1 mutants, even when they cannot be degraded by Vpx. HEK293T was transfected as indicated and treated with 10 mM MG132 for 12 h before collection. Subsequently, co-IP using anti-HA antibodies and IB analyses were performed. (**E**) USP37 deubiquitinated both K48- and K63-linked ubiquitin chains of SAMHD1 △HD domain. (**F**) USP37 can interact with SAMHD1 WT, ΔSAM, and ΔHD mutants through co-IP analysis. (**G, H**) *In vitro* deubiquitination assay. Ubiquitinated SAMHD1 was purified from HEK293T cells transfected with Ub-Flag, Vpx-myc, or TRIM21-V5 and SAMHD1-HA using anti-HA affinity purification. USP37 341-979 recombinant protein was purified by Ni2+-NTA beads. Ubiquitinated SAMHD1-HA was incubated with His-tagged USP37 341-979 for 12 h at 37°C, then it was analyzed using IB or SDS–PAGE and visualized with Coomassie staining.

To validate the differences in USP37-mediated deubiquitination of SAMHD1 mutants, we constructed the deletion mutants of SAMHD1 (Fig. 5A). SAMHD1 △SAM and △NLS mutants could be degraded by Vpx, while the SAMHD1△HD and 1–547 mutants could not be degraded (Fig. 5B). Interestingly, USP37 stabilized SAMHD1 WT and all mutants, even the △HD and △NLS mutants resistant to Vpx degradation (Fig. 5B). For further verification, we selected two representatives of △SAM that could be degraded by Vpx and △HD mutants that could not be degraded by Vpx. In the absence of Vpx, SAMHD1 WT, △SAM, and △HD also could be ubiquitinated, while the △HD mutant showed the strongest ubiquitination ability (Fig. 5C, lane 4). This is consistent with our previous research showing that SAMHD1 is also degraded by E3 ligase TRIM21, in addition to Vpx-induced degradation (16). Therefore, USP37 could remove the ubiquitination of SAMHD1 WT, ΔSAM, and ΔHD (Fig. 5D, lanes 5–7). Furthermore, USP37 removed both K48 and K63 polyubiquitin from the SAMHD1 ΔHD mutant resistant to Vpx degradation (Fig. 5E). Additionally, USP37 showed the ability to bind to the SAMHD1 WT, ΔHD mutant, and ΔSAM (Fig. 5F). *In vitro* deubiquitination analysis showed that polyubiquitinated SAMHD1, whether induced by Vpx or TRIM21, could be deubiquitinated by USP37 purified *in vitro* (Fig. 5G and H). Overall, USP37 deubiquitinates SAMHD1 by recognizing SAMHD1 rather than recognizing the E3 complex.

### Deubiquitinase activity and UIM motifs of USP37 are required for SAMHD1 deubiquitination

**Fig 6:**
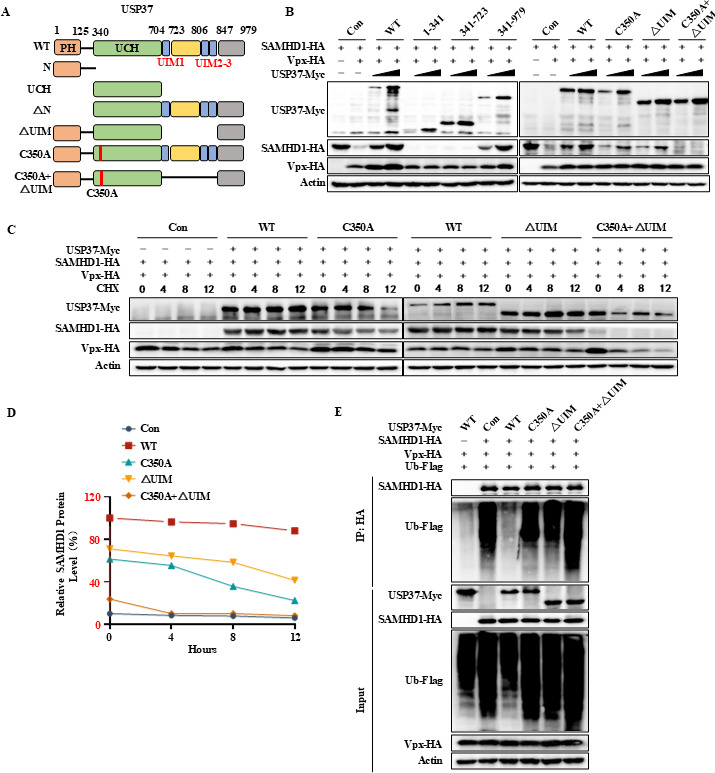
Deubiquitinase activity and UIM motifs of USP37 are required for SAMHD1 deubiquitination. (**A**) Schematic representation of USP37 wild type and mutants used in the study. (**B**) Effects of different USP37 mutants on Vpx-induced degradation of SAMHD1. (**C, D**) HEK 293T cells were transfected with Vpx-HA, SAMHD1-HA, and USP37-Myc WT or mutants, and then treated with CHX (50 µg/mL) before the cell harvesting was performed. Cells were harvested at the indicated times, and protein levels were determined by immunoblotting (**C**). SAMHD1 expression was quantified using ImageJ with control actin (D). (**E**) The deletion of USP37 enzyme activity and UIM jointly impairs the deubiquitination of SAMHD1. HEK293T cells were transfected as indicated, followed by treatment with 10 mM MG132 for 12 h prior to collection. Cell lysates were co-IP with anti-HA antibodies conjugated to agarose beads. Cell lysates and precipitated samples were analyzed by immunoblotting with the corresponding antibodies.

USP37 consists of an N-terminal pleckstrin homology (PH) domain, a central ubiquitin carboxyl-terminal hydrolase (UCH) domain, and a C-terminal ubiquitin-interacting motif (UIM motif) (Fig. 6A). To determine which domain of USP37 is essential for the deubiquitination and stabilization of SAMHD1, we generated truncated and deubiquitinase activity-deficient mutants of USP37 (USP37 C350A) (Fig. 6A). The USP37 1-341, 341-723, and C350A + △UIM mutants lost the ability to stabilize SAMHD1 (Fig. 6B), indicating the UCH and UIM domains required for the deubiquitination. CHX assay showed that USP37 C350A + ΔUIM mutant lost the ability to prolong the half-life of SAMHD1 as the negative control group (Fig. 6C and D). *In vivo* deubiquitination assay indicated that the USP37 C350A and △UIM single mutations still maintained partial deubiquitinase activity, while the USP37 C350A + △UIM double mutant completely lost its deubiquitination ability (Fig. 6E). In summary, the UCH domain and UIM motifs of USP37 are essential for the deubiquitination of SAMHD1.

### The phosphorylation activity-deficient mutant of USP37 does not affect its deubiquitination ability

**Fig 7:**
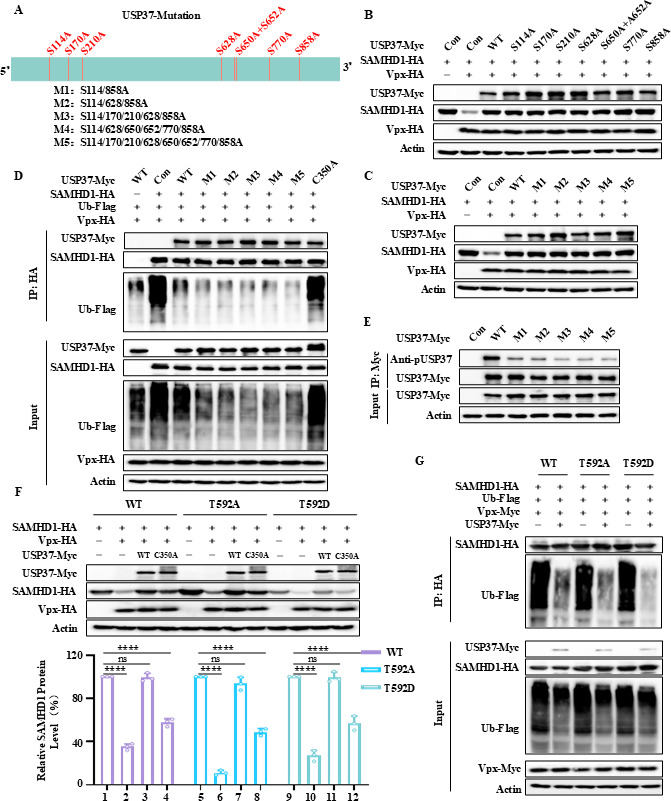
The phosphorylation activity-deficient mutant of USP37 does not affect its deubiquitination ability. (**A**) Schematic representation of USP37 phosphorylation site mutants used in the study. (**B, C**) Effects of USP37 phosphorylation activity-deficient mutations on Vpx-induced degradation of SAMHD1. (**D**) USP37 phosphorylation activity-deficient mutations can remove polyubiquitination from SAMHD1. HEK293T cells, transfected as instructed, were treated with 10 mM MG132 for 12 h before collection. Subsequently, co-IP using anti-Myc antibodies and IB analyses were performed. (**E**) The phosphorylation level of USP37 and mutation were detected by co-IP and immunoblotting. (**F**) USP37 can restore the degradation of SAMHD1 T592A and T592D by Vpx. Transfections were performed as indicated. After 48 h, cells were harvested, and protein expression was analyzed by IB (above). SAMHD1 expression was quantified using ImageJ with control actin (below). (**G**) USP37 removed polyubiquitination of SAMHD1 T592A and T592D. Transfections were performed as indicated, and cells were treated with 10 mM MG132 12 h before harvesting. Cell lysates were co-IP with anti-HA antibodies coupled to agarose beads. Cell lysates and precipitation samples were analyzed by immunoblotting and corresponding antibodies. Actin was used as the loading control for the cell lysate. T592A: phosphorylation deletion mutant; T592D: phosphorylation mimic mutant. Data are means ± SD from *n* = 3 independent experiments. Statistical significance was analyzed using two-sided unpaired *t*-tests (NS, not significant; ****, *P* < 0.0001).

Phosphorylation regulates the enzymatic activities of DUBs (27). Next, we investigated whether USP37 phosphorylation affects its ability to remove ubiquitination of SAMHD1. We mutated all reported serine phosphorylation sites of USP37 to alanine, either as single-point mutations or in combination (Fig. 7A). The results indicated that single or combined mutations in USP37 did not impair its ability to stabilize SAMHD1 (Fig. 7B and C). Deubiquitination assays further demonstrated that USP37 mutants lacking phosphorylation, similar to WT, could remove polyubiquitin chains from SAMHD1, whereas the C350 catalytic activity-deficient mutant lost its deubiquitination ability (Fig. 7D). Western blot analysis showed that the phosphorylation-deficient mutant of USP37 was almost non-phosphorylatable (Fig. 7E). Therefore, the phosphorylation of USP37 does not affect its deubiquitination activity toward SAMHD1.

A previous study indicated that USP37 enhances the stability of phosphorylated Cdt1 via deubiquitination (28). To investigate whether the deubiquitination activity of USP37 is affected by substrate SAMHD1 phosphorylation, we constructed SAMHD1 phosphorylation-defective T592A and phosphomimetic T592D mutants. The results showed that USP37 inhibited the degradation of SAMHD1 T592A and T592D induced by Vpx, whereas the USP37 C350A mutant exhibited a significant reduction in the stabilization of SAMHD1 (Fig. 7F). The deubiquitination assay also demonstrated that USP37 deubiquitinated both SAMHD1 T592A and T592D (Fig. 7G). These findings suggest that phosphorylation of SAMHD1 does not affect the ability of USP37 to deubiquitinate SAMHD1.

### USP37 inhibits SIV infection and LINE1 retro-transposition activity by stabilizing SAMHD1

**Fig 8:**
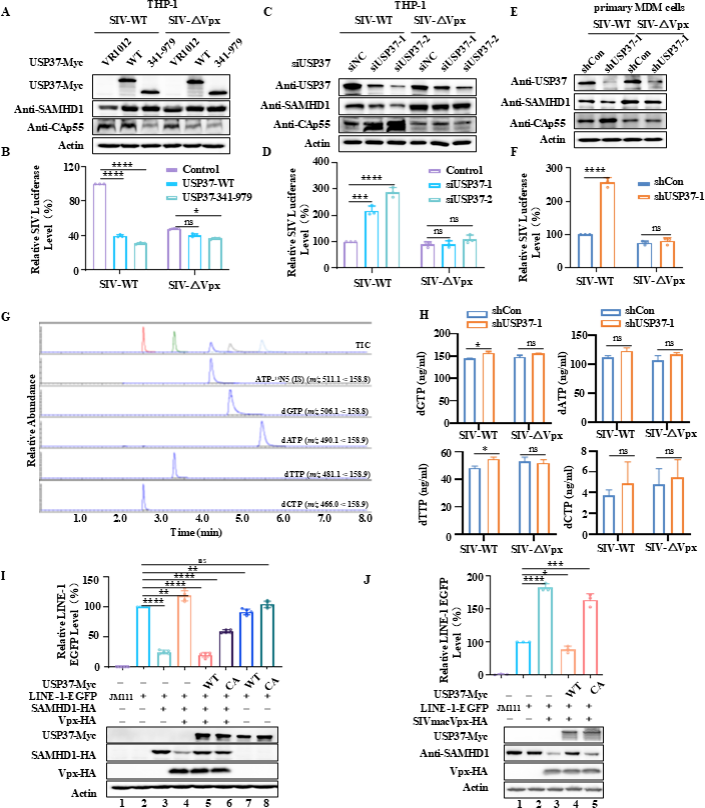
USP37 inhibits SIV infection and LINE1 retrotransposition activity by stabilizing SAMHD1. (**A–D**) Effect of overexpression or knocked down USP37 on SIV infectivity. (**A**) USP37 WT and USP37 341-979 (the catalytic core truncation of USP37) or control vector was electro-transfected into THP-1. (**B**) USP37 was knocked down in THP-1. Then, THP-1 cells were differentiated into macrophages by 0.1 mM PMA treatment and infected with SIVmac239-WT or SIVmac239-△Vpx viruses for 12 h. Subsequently, the cells were washed thrice with phosphate-buffered saline (PBS) and placed in fresh RPMI-1640 medium with 10% fetal bovine serum. The cells and supernatants were harvested after 48 of infection. (**A, C**) Cell lysates were analyzed by immunoblotting. (**B, D**) Virus infectivity was assessed using TZM-BL indicator cells. (**E, F**) After human monocyte-derived macrophages (MDM) cells were induced to differentiate, they were infected with shUSP37 lentivirus. After 48 h, the medium was changed, and the cells were infected with -WT or △Vpx viruses. The virus was removed after 12 h, and 48 h later, cells and viral supernatants were collected. The cells were used for western blot analysis and dNTP extraction. (**F**) The viral load in the supernatants was measured using TZM-BL indicator cells. (**G**) The chromatograms of the internal standard ATP-^15^N5 and dNTP standards were analyzed using the high-performance liquid chromatography–tandem mass spectrometry (HPLC–MS/MS) method. (**H**) The HPLC–MS/MS method was used to analyze the dGTP, dATP, dTTP, and dCTP levels in primary MDM cells. (**I, J**) USP37 affects the ability of SAMHD1 to inhibit LINE1. HEK293T cells were transfected with the LINE-1-EGFP reporter plasmid or the control vector JM111, together with empty vector, USP37-Myc and USP37 C350A-Myc (USP37 CA), Vpx-HA, and SAMHD1-HA(C)/endogenous SAMHD1(D). After 5 days, EGFP-positive cells were isolated using flow cytometry (upper). Western blotting shows the protein levels of USP37, SAMHD1, and Vpx (bottom). Data are means ± SD from *n* = 3 independent experiments. Statistical significance was analyzed using two-sided unpaired *t*-tests (NS, not significant, *P < 0.05, **P < 0.01; ***P < 0.001, and ****P < 0.0001).

SAMHD1 is a multifunctional protein involved in various biological processes, including limiting viral replication, inhibiting retrotransposon activity, regulating the cell cycle, and immune modulation (9, 17, 29, 30). To investigate how USP37 affects the function of SAMHD1, we knocked down or overexpressed USP37 in THP-1 cells, the target cells for SIV infection, and examined its impact on the infection of SIVmac239 WT or SIVmac239 ΔVpx. The results showed that overexpressing USP37 WT and the catalytic center USP37 (USP37-341-979) could effectively inhibit the production of SIVmac239 WT but has no significant effect on the production of SIVmac239 △Vpx (Fig. 8A and B). Conversely, USP37-knockdown increased the production of SIVmac239 WT, but it had no significant effect on the production of SIVmac239 △Vpx (Fig. 8C and D), indicating that USP37 suppresses the replication of SIVmac239 WT through inhibiting the Vpx-mediated degradation of SAMHD1. A similar result was also determined in primary MDM cells (Fig. 8E and F). Previous reports have shown that SAMHD1 can regulate intracellular dNTP pools in monocyte-derived macrophages through its dNTPase activity, thereby inhibiting the replication of retroviruses (11). In this study, we also analyzed changes in intracellular dNTP levels using the HPLC–MS/MS method. The detection of dNTP and internal standards (ATP-^15^N5) confirmed that the experimental method was reliable (Fig. 8G). The results showed that when USP37 was knocked down, and cells were infected with SIVmac239 WT, the levels of dGTP and dTTP increased, while those of dATP and dCTP showed no significant changes. Additionally, in cells infected with SIVmac239 △Vpx, there were no changes in the levels of dGTP, dTTP, dATP, or dCTP (Fig. 8H).

SAMHD1 inhibits the transposition activity of LINE1 (17). To test whether USP37 affects the ability of SAMHD1 to inhibit LINE1, a well-established reporter system was used to evaluate the effect of SAMHD1 on LINE-1 retro-transposition. JM111, which contains a double mutation of R261A/R262A in the ORF1, served as a negative control for LINE-1-EGFP transcriptional activity (17, 31). The LINE-1-EGFP reporter plasmid, SIVmacVpx-HA, USP37 WT, or C350A mutant plasmids were co-transfected into HEK293T cells, and LINE1-EGFP-positive cells were quantified 5 days after transfection using flow cytometry. The results showed that SAMHD1 inhibited LINE1 activity, whereas the addition of Vpx significantly weakened the SAMHD1 inhibitory effect on LINE1 (Fig. 8I, lanes 3 and 4). However, USP37 WT restored the ability of SAMHD1 to inhibit the LINE1 activity, but the C350A mutant only partially restored the SAMHD1 activity (Fig. 8I, lanes 5 and 6). These results correspond to data showing that USP37 C350A partially lost its ability to inhibit Vpx-mediated degradation of SAMHD1 (Fig. 6). Further analysis showed that USP37 restored the inhibitory effect of Vpx on endogenous SAMHD1 (Fig. 8J). In summary, USP37 enhances antiviral and anti-retrotransposition activities by stabilizing SAMHD1.

## DISCUSSION

SAMHD1 is a potential host factor that can regulate the replication of multiple viruses (16, 30) (32). Mutations in SAMHD1 are associated with Aicardi–Goutières syndrome, a rare genetic autoimmune disorder characterized by chronic brain inflammation and abnormal activation of the immune system, resulting in symptoms that resemble viral infections (33). SAMHD1 also plays a role in tumor development and progression by modulating the cell cycle and DNA damage repair mechanisms (34). Overall, SAMHD1 is a key protein involved in antiviral defense, immune regulation, and cell growth control. Therefore, modulating SAMHD1 expression and activity represents a promising therapeutic strategy for a range of diseases.

Ubiquitination and deubiquitination maintain dynamic equilibrium within organisms. Deubiquitination is a reversible process of protein ubiquitination that plays a crucial role in maintaining cellular homeostasis (35). Both E3 ubiquitin ligases and deubiquitinases (DUBs), which are involved in these processes, are gaining recognition as promising targets for drug discovery. For example, the E3 ligase RNF5-mediated degradation of envelope (E) protein curtails severe acute respiratory syndrome–coronavirus 2 (SARS–CoV-2) replication. However, Analog-1, a pharmacological activator of RNF5, suppresses SARS–CoV-2 virulence by facilitating the degradation of the E protein, highlighting RNF5 targeting as an effective broad-spectrum antiviral strategy. Conversely, deubiquitinases have also been considered drug targets in many studies. For example, USP1 removes ubiquitination from the SARS–CoV-2 non-structural protein ORF6, thereby enhancing viral replication. Nevertheless, the USP1 inhibitor ML323 specifically blocks the USP1 activity, effectively inhibiting viral replication. USP9X regulates the cell cycle, protein endocytosis, apoptosis, cell polarity, immune microenvironment, and stem cell traits. WP1130 and its analog EOAI3402143 (G9) can effectively inhibit the activity of USP9X, thereby inhibiting the progression of many tumors (36–39). In this study, we demonstrated that deubiquitinase USP37 effectively inhibits the Vpx-mediated degradation and antiviral and anti-retrotransposition activities of SAMHD1 (Fig. 1, 2 and 8). This suggests that we can screen USP37 activators that further stabilize SAMHD1, thereby enhancing its function.

Our results also showed that USP37 interacted with SAMHD1 but not with the E3 complex recruited by Vpx (Fig. 4). Therefore, the stabilization of SAMHD1 by USP37 is due to the recognition of SAMHD1 itself and not the E3 complex. Interestingly, USP37 could enhanced the stability of SAMHD1 in the absence of Vpx or SAMHD1 mutant resistance to Vpx-mediated degradation (Fig. 5B). This can be explained by our previous research that SAMHD1 also is degraded by TRIM21 recruited by EV71 viruses (16). We further demonstrated that USP37 can deubiquitinate the polyubiquitin of SAMHD1 induced by TRIM21 (Fig. 5). *In vitro* deubiquitination analysis showed that the polyubiquitination of SAMHD1, whether induced by Vpx or TRIM21, could be deubiquitinated by USP37 purified in *Escherichia coli* (Fig. 5G and H).

Post-translational modifications (PTMs) significantly affect protein function in various ways. Modifications, such as phosphorylation, acetylation, ubiquitination, and methylation, can alter protein structure, localization, stability, and interactions with other molecules (40). For example, phosphorylation can activate or deactivate enzymes, modify signaling pathways, and affect gene expression (41, 42). Cdk2-dependent phosphorylation enhanced the activity of USP37 toward cyclin A. However, the phosphorylation-deficient S628A mutant of USP37 is inactive (43). Here, we mutated all critical phosphorylation sites on USP37 to prevent its phosphorylation and found that USP37 WT and phosphorylation-deficient mutants showed no significant difference in reversing Vpx-mediated SAMHD1 degradation (Fig. 7).

In conclusion, we have discovered that USP37 extensively inhibited the Vpx-mediated degradation of SAMHD1 across various HIV-2/SIV subtypes. USP37 stabilizes SAMHD1 through interactions and removal of ubiquitination. Deubiquitinase activity and UIM motifs of USP37 are required for SAMHD1 deubiquitination, but the phosphorylation state of USP37 does not affect its deubiquitination activity. Furthermore, USP37 enhances antiviral efficacy and suppresses retrotransposition of LINE-1 elements by stabilizing SAMHD1. Our findings provide evidence that enhancing the antiviral activity of DUBs is a viable strategy against HIV/SIV infections.

## MATERIALS AND METHODS

### Plasmid construction

All USPs with HA-Flag tag were purchased from Addgene (Watertown, MA, USA). USP37-HA was constructed with a C-terminal HA tag and inserted between the SalI and BamHI sites of VR1012. The USP37 gene with a C-terminal Myc tag was amplified using USP37-HA as the template and was cloned into the VR1012-Entry vector via the SalI and BamHI sites. The phosphorylation activity-deficient and truncating mutations of USP37 were constructed with USP37-Myc by PCR-targeted mutagenesis. The primers for all mutations are given in Table 1. The expression vectors pSIVmac239 Vpx-HA (44), HIV-2_Rod_Vpx (45), and SAMHD1-HA (45) were constructed as described previously. The expression vectors SAMHD1-T592A, SAMHD1-T592D, SAMHD1 Δ42–108, and SAMHD1 Δ162–335 were obtained from the Institute of Virology and AIDS Research, First Hospital of Jilin University and were as previously described (45, 46).

**TABLE 1:** Primers used in this study

Primer name	Primer direction	Sequence (5′–3′)
USP37-S114A	F	CAGCAGGCAGCTTGCATACTCAGACAATCAG
	R	TGCAAGCTGCCTGCTGGTTTCCTTC
USP37-S170A	F	TCCGGGTAGAGGAGCGATTAAGACTGTAGCAG
	R	CTCCTCTACCCGGATTACCAAG
USP37-S210A	F	GAGGAAAAGAATGATAGCAACTGGCTCAGAATTG
	R	CTATCATTCTTTTCCTCTTTTCAGTAC
USP37-C350A	F	CAATTTGGGAAATACCGCCTATATGAATGCTATTC
	R	GCGGTATTTCCCAAATTGGAGAAGCCCTG
USP37-S628A	F	GTGAATTCCTGCATCACAGCCCCTTCTACACCTTC
	R	TGTGATGCAGGAATTCACCATTTGAGAG
USP37-S650A + S652A	F	GCTTTATGCCTTGATGCAGACGCTGAGGATGAGCTAAAAC
	R	TGCATCAAGGCATAAAGCCAAGGAGCTC
USP37-S770A	F	GCTGGATCCTGCCGCTTTTACTGAGATAACTAAA
	R	GCGGCAGGATCCAGCTCTGTGATTG
USP37-S858A	F	GGTTCTGATGAGGACGCTGGAAATGAGGATGTTTTTG
	R	AGCGTCCTCATCAGAACCCAATGCATCC
USP37-1-340	F(Myc)	CGACACGTGTGATCAGATATGGAACAAAAACTTATCTCAGAAGAAGATCTCTCTCCTCTGAAGATACAT
	R	GTCTAGAGCGGCCGCGATTCACAGTTGCTGCTGTTGG
USP37-341-723	F(Myc)	CGACACGTGTGATCAGATATGGAACAAAAACTTATCTCAGAGGAAGATCTTCAGGGCTTCTCCAATTTG
	R	GTCTAGAGCGGCCGCGATTCAAGATGGTGAAGCATCTCTCTTACTTATC
USP37-341-979	F(Myc)	CGACACGTGTGATCAGATATGGAACAAAAACTTATCTCAGAGGAAGATCTTCAGGGCTTCTCCAATTTG
	R	GTCTAGAGCGGCCGCGATTCAGGTAGTCTTCCCCACTTC
USP37-Δ704–847	F	AGGATTTGACAGAATGTTTGTGGATGCATTG
	R	AACATTCTGTCAAATCCTGAGTTTTCC
shUSP37-1	F	CCGGCAGCTAAGTCATAACATTACTCGAGTAATGTTATGACTTAGCTGTTTTTG
	R	AATTCAAAAACAGCTAAGTCATAACATTACTCGAGTAATGTTATGACTTAGCTG
shUSP37-2	F	CCGGCCAAGGATATTTCAGCTAACTCGAGTTAGCTGAAATATCCTTGGTTTTTG
	R	AATTCAAAAACCAAGGATATTTCAGCTAACTCGAGTTAGCTGAAATATCCTTGG
shSAMHD1	F	CCGGAATGTACACGCATGCTGAAGCCTCGAGGCTTCAGCATGCGTGTACATTTTTTTG
	R	AATTCAAAAAAATGTACACGCATGCTGAAGCCTCGAGGCTTCAGCATGCGTGTACATT
GAPDH-RT	F	GCAAATTCCATGGCACCGT
	R	TCGCCCCACTTGATTTTGG
USP37-RT	F	GGAACACTCTTCTGGTGGCA
	R	GTGAGAGGAAAGGGGCACTC

### Cells

HEK293T (CRL-11268; ATCC, Manassas, VA, USA) and TZM-bl (PTA-5659; ATCC) cells were maintained in Dulbecco’s modified Eagle’s medium (DMEM; HyClone, South Logan, UT, USA) containing 10% heat-inactivated fetal bovine serum (04-001-1; Biological Industries, Kibbutz Beit Haemek, Israel) and 100 µg/mL penicillin–streptomycin. Human monocytic cell line THP-1 (TIB-202; ATCC) cells were maintained in RPMI 1640 medium (RPMI 1640; HyClone) containing 10% fetal bovine serum and 100 µg/mL penicillin–streptomycin. All cells were placed in a humidified atmosphere at 37°C in an incubator containing 5% (vol/vol) CO_2_.

### Transfection

DNA transfection into HEK293T was carried out using Lipofectamine 2000 (52887; Invitrogen, Carlsbad, CA, USA) according to the manufacturer’s instructions. THP-1 cells were transfected using the recommended kit (s)-SG Cell Line 4D-Nucleofector™ X Kit (Lonza, Switzerland) with the program DV-100 according to the manufacturer’s instructions.

### SIV infectivity and detection

For the SIV-like particle-containing packaging assay, HEK293T cells prepared in six-well plates were co-transfected with 2 µg of SIVmac239 WT or SIVmac239 ΔVpx expression vectors together with 0.4 µg of pCMV encoding VSV-G. After 6 h, the supernatant was removed and supplemented with 2 mL of fresh DMEM medium containing 10% FBS. 48 h after transfection, the supernatant was collected and filtered through a 0.45 mm filter. Then, THP-1 cells were infected with SIVmac239 WT or SIVmac239 ΔVpx for 12 h. Subsequently, the cells were washed thrice with phosphate-buffered saline (PBS) and placed in fresh RPMI-1640 medium with 10% fetal bovine serum (FBS). The culture medium was harvested 48 h after infection. SIV infectivity was assessed using TZM-BL indicator cells. LTR-luciferase was activated when TZM-BL cells were infected by SIV. TZM-BL cells were seeded in 24-well format plates (2 × 10^4^ cells/well). Then, 24 h later, cells were infected with a total volume of 30 µL of viral dilutions for 48 h under 20 µg/mL DEAE conditions. A dual-luciferase reporter gene detection system (E2810; Promega, Madison, WI, USA) was used according to the manufacturer’s protocol using a GloMax 20/20 light meter (Promega).

### Reagents and antibodies

The antibodies used in this study were as follows: anti-actin monoclonal antibody (A00702-100; GenScript, Piscataway, New Jersey, USA), anti-HA monoclonal antibody (901514; BioLegend, Peking, China), anti-Myc monoclonal antibody (AHO0052; Invitrogen, Carlsbad, CA, USA), anti-Flag mAb (F1804, Sigma-Aldrich, St. Louis, MO, USA), anti-CAp24 monoclonal antibody (1513; AIDS Research and Reference Reagents Program, Division of AID, NIAID, NIH), anti-SAMHD1 (12586-1-AP; Proteintech, Wuhan, Hubei Province, China), anti-USP37 (18465-1-AP; Proteintech, Wuhan, Hubei Province, China), and anti-phospho-threonine (PTM-705RM, PtmBio, Hangzhou, Zhejiang Province, China). Secondary antibodies were alkaline phosphatase-coupled anti-rabbit (111-035-045; Jackson, West Grove, PA, USA) and anti-mouse (115-035-062; Jackson, West Grove, PA, USA). All antibodies were used according to the manufacturer’s protocol.

### Immunoblot analysis

For the immunoblotting (IB) analysis of cell-associated proteins, expression plasmids were transfected into HEK293T or THP-1 cells. Then, 48 h after transfection, cells were collected in culture medium and centrifuged at 7000×*g* for 5 min. Cells were mixed with appropriate volumes of lysis buffer (50 mM Tris–HCl, pH 7.8, 150 mM NaCl, 1% NP-40, 1% sodium deoxycholate, and 4 mM EDTA) and 4× sampling buffer (0.08 M Tris–HCl, pH 6.8, 2.0% SDS, 10% glycerol, 0.1 M dithiothreitol, and 0.2% bromophenol blue). The mixture was heated at 100°C for 30 min and vortexed appropriately to shear cellular DNA. Proteins were separated by SDS–PAGE. Proteins were transferred to a nitrocellulose membrane (10401396; GE Whatman, Shanghai, China) and treated with a sealant solution for 20 min after incubation with appropriate antibodies. The membranes were subsequently incubated with horseradish peroxidase-coupled secondary antibodies, and proteins on the PVDF membranes were visualized by using the Ultra-Sensitive ECL Chemiluminescence Detection Kit (B500024; Proteintech). ImageJ software was used to calculate signal values for western blotting data.

### Co-immunoprecipitation

HEK293T cells were transfected with the appropriate plasmids according to experimental instructions. Cells were treated with 10 mM MG132 (S2619; Selleck, Houston, TX, USA) 12 h before harvesting, and after harvesting, cells were washed twice with cold PBS, followed by lysis with 1 mL of lysing solution (50 mM Tris, pH 7.5, 150 mM NaCl, 1% NP-40, and Complete Protease Inhibitor Mixture Tablets (11836170001; Roche, Basel, Switzerland)) at 4°C for 1 h of lysis. Cell lysates were clarified by centrifugation at 12,000×*g* for 30 min at 4°C. Protein G-agarose (11243233001; Roche, Basel, Switzerland) was preincubated with anti-HA antibody, anti-Myc antibody, or anti-Flag antibody for 1 h before use. Subsequently, the preincubated protein G-agarose was mixed with the prepared cell lysate and incubated at 4°C for 3 h or overnight. Post-reaction mixtures were washed with cold wash buffer (20 mM Tris, pH 7.5, 100 mM NaCl, 0.1 mM EDTA, and 0.05% Tween 20) for six times, and the samples were centrifuged for 1 min each time at 800×*g* at 4°C. Proteins were eluted with elution buffer (0.1 M glycine–HCl, pH 2.5) and analyzed using SDS–PAGE and immunoblotting.

### RT-qPCR

RNA was isolated with TRIzol reagent by following the manufacturer’s instructions (15596-026; Invitrogen, Carlsbad, CA, USA). According to the manufacturer’s instructions, RNA reverse transcription was performed using the EasyScript First-strand cDNA Synthesis SuperMix (AE301; TransGen Biotech, Beijing, China). Each cDNA synthesis reaction used 250 to 1,000 ng of total RNA as a template, with blank samples containing only H_2_O or no reverse transcriptase. FastStart Universal SYBR Green Master (Rox) (491314001; Roche, Basel, Switzerland) was used as the fluorescent nucleic acid dye. Data were normalized to the housekeeping gene GAPDH, and the relative abundance of the target gene was calculated using the Ct model. The primers used in this study are listed in Table 1.

### Knockdown cell line construction

A specific shRNA with the target site USP37/SAMHD1 was cloned in the lentiviral vector pLKO.1-puro (Addgene). Lipofectamine 2000 (52887; Invitrogen, Carlsbad, CA, USA) co-transfected HEK293T cells with sh-USP37/SAMHD1-pLKO.1 or pLKO.1 with RRE, REV, and VSV-G expression vectors. After 48 h of transfection, the supernatant containing the packaged lentivirus is harvested and used to infect HEK293T or THP-1 cells for 48 h. It was then treated with a solution containing puromycin (P8833; Sigma, St. Louis, MO, USA) (HEK293T 5 µg/mL, THP-1 2.5 µg/mL) to screen stable cell lines. shRNA primers are listed in Table 1.

### Human monocyte-derived macrophage isolation and differentiation

Peripheral blood mononuclear cells were isolated from healthy donors (after obtaining informed consent) using density gradient centrifugation (Ficoll-Hypaque: 17144003, Cytiva, Uppsala, Sweden). The cells were cultured in RPMI 1640 medium supplemented with 10% human AB serum (100-512; GeminiBio, West Sacramento, CA, USA) for 4 h, and nonadherent cells were discarded. The adherent monocyte-derived macrophages (MDMs) were then cultured in RPMI 1640 medium supplemented with 10% human AB serum and 15 ng/mL M-CSF (HY-P7050; MedChem Express, Monmouth Junction, NJ, USA) at 37°C with 5% CO2 for 4 days.

### Quantification of intracellular dNTP levels by HPLC–MS/MS

dNTPs were extracted from primary macrophages (2 × 10⁶ cells) that were USP37 knockout and infected with recombinant SIVmac239 WT or ΔVpx. Cells were treated with 60% ice-cold methanol and incubated at 95°C for 5 min. The samples were vortexed, sonicated on ice for 30 s, then centrifuged at 13,000×*g* at 4°C for 20 min. The supernatant was collected and transferred to a new 1.5 mL EP tube, stored at −80°C, and later injected into the HPLC–MS/MS system for analysis.

dNTPs in cellular samples were quantified using a rapid HPLC-MS/MS method. After extraction, samples were vortexed, mixed with an internal standard (500 nM) and mobile phase A (100 mM ammonium acetate, pH 10.0) in a 100:10:90 ratio, vortexed again, centrifuged, and analyzed on a porous graphitized carbon column. Separation was achieved with a Shimadzu LC-40AD system and AB SCIEX QTRAP 6500+ mass spectrometer. Chromatography was performed using a Hypercarb column (2.1 mm × 50 mm, 3 µm) with solvents A (0.1 M ammonium acetate, pH 10.0) and B (0.1% ammonium hydroxide in acetonitrile) at a 0.3 mL/min flow rate. The gradient and runtime were optimized to separate dCTP, dTTP, dGTP, and dATP with retention times of 2.51, 3.27, 4.74, and 5.53 min, respectively. The retention time for the internal standard (IS), ATP-^15^N5, was 4.16 min. Analytes were monitored in negative ion mode using electrospray ionization (ESI), and MRM transitions were m/z 466.0 > 158.9 for dCTP, 481.0 > 158.9 for dTTP, 506.1 > 158.9 for dGTP, and 490.1 > 158.9 for dATP. Data were processed with Analyst 1.7 software, and chromatographic conditions were optimized to resolve overlapping peaks, including an initial misidentification of ATP-^15^N5 as dGTP.

### Recombinant protein expression and puriﬁcation

For expression, the plasmids were transformed into *E. coli* BL21(DE3) cells. The proteins were over-expressed overnight at 20℃ by induction with 0.5 mM isopropyl-D-thiogalactopyranoside. Harvested cells were lysed in 20 mM Tris–HCl, pH 8.0, with 200 mM NaCl, and then clarified by sonication and centrifugation at 13,000×*g* for 30 min. The supernatant was transferred to NiNTA beads (C600033, BBI, Shanghai, China), and the flow through was loaded onto NiNTA beads for two more passages. After washing with 20 mM Tris–HCl, pH 8.0, with 200 mM NaCl and 100 mM imidazole, the protein was eluted with 20 mM Tris–HCl, pH 8.0, with 200 mM NaCl and 250 mM imidazole.

### *In vitro* deubiquitination assay

Ubiquitinated SAMHD1 was isolated from HEK293T cells transfected with expression vectors of Ub-Flag, Vpx-Myc, and SAMHD1-HA (or TRIM21-V5), and then puriﬁed from the cell extracts with anti-HA or anti-V5 antibody conjugated protein G agarose beads. USP37 fusion protein was puriﬁed by metal-afﬁnity chromatography on chelation resin (NiNTA beads). For *in vitro* deubiquitination assay, ubiquitinated SAMHD1 protein was incubated with USP37 in the deubiquitination buffer (20 mM Tris–HCl pH 8.0, 200 mM NaCl, 1 mM EDTA, 10 mM DTT, 5% glycerol) for 1 h at 37°C. The ubiquitinated SAMHD1 was analyzed by immunoblotting.

### Statistical analysis

All data represent the results of three independent experiments and are presented as the mean ± standard deviation (SD). Differences among groups were analyzed using ANOVA. NS, not significant; *P < 0.05; **P < 0.01; ***P < 0.001; and ****P < 0.0001.

## Data Availability

All data and reagents are available upon request.
